# Supplementary material for: Causal Relationship between Aging and Anorexia Nervosa: A White-Matter-Microstructure-Mediated Mendelian Randomization Analysis
Source: Biomedicines. 2024 Aug 16;12(8):1874. doi: 10.3390/biomedicines12081874 (PMC11351342; doi:10.3390/biomedicines12081874)
Supplement: Supplementary file 1 [file biomedicines-12-01874-s001.zip › Supplementary Figure/Figure S4/Singe SNP plot of MR analysis for TL to ubm-b-1847.pdf]

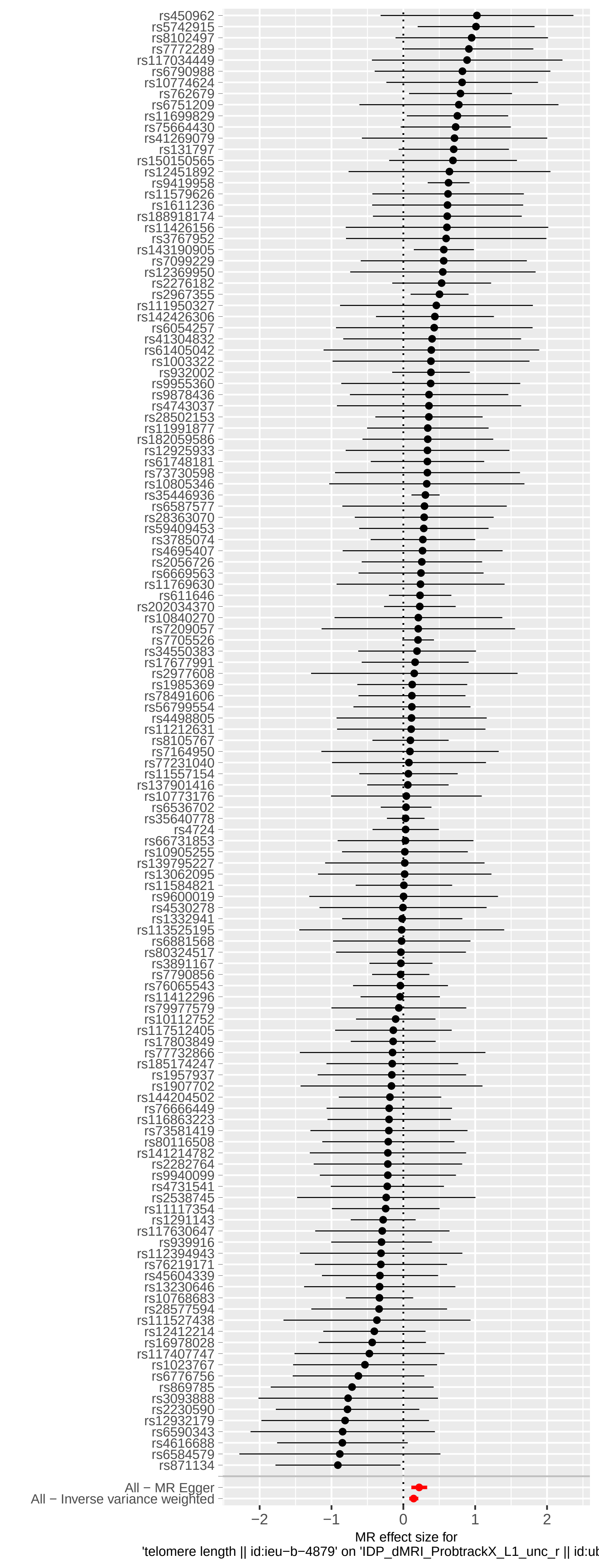
rs450962
rs5742915
rs8102497
rs7772289
rs117034449
rs6790988
rs10774624
rs762679
rs6751209
rs11699829
rs75664430
rs41269079
rs131797
rs150150565
rs12451892
rs9419958
rs11579626
rs1611236
rs188918174
rs11426156
rs3767952
rs143190905
rs7099229
rs12369950
rs2276182
rs2967355
rs111950327
rs142426306
rs6054257
rs41304832
rs61405042
rs1003322
rs932002
rs9955360
rs9878436
rs4743037
rs28502153
rs11991877
rs182059586
rs12925933
rs61748181
rs73730598
rs10805346
rs35446936
rs6587577
rs28363070
rs59409453
rs3785074
rs4695407
rs2056726
rs6669563
rs11769630
rs611646
rs202034370
rs10840270
rs7209057
rs7705526
rs34550383
rs17677991
rs2977608
rs1985369
rs78491606
rs56799554
rs4498805
rs11212631
rs8105767
rs7164950
rs77231040
rs11557154
rs137901416
rs10773176
rs6536702
rs35640778
rs4724
rs66731853
rs10905255
rs139795227
rs13062095
rs11584821
rs9600019
rs4530278
rs1332941
rs113525195
rs6881568
rs80324517
rs3891167
rs7790856
rs76065543
rs11412296
rs79977579
rs10112752
rs117512405
rs17803849
rs77732866
rs185174247
rs1957937
rs1907702
rs144204502
rs76666449
rs116863223
rs73581419
rs80116508
rs141214782
rs2282764
rs9940099
rs4731541
rs2538745
rs11117354
rs1291143
rs117630647
rs939916
rs112394943
rs76219171
rs45604339
rs13230646
rs10768683
rs28577594
rs111527438
rs12412214
rs16978028
rs117407747
rs1023767
rs6776756
rs869785
rs3093888
rs2230590
rs12932179
rs6590343
rs4616688
rs6584579
rs871134
All − MR Egger
All − Inverse variance weighted
−2
−1
0
1
2
MR effect size for
'telomere length || id:ieu-b-4879' on 'IDP_dMRI_ProbtrackX_L1_unc_r || id:ub
